# Spontaneous Retroperitoneal Hematoma: A Deadly Complication for Patients Awaiting Liver Transplant

**DOI:** 10.7759/cureus.32522

**Published:** 2022-12-14

**Authors:** Badi Rawashdeh, Joohyun Kim, Johnny Hong

**Affiliations:** 1 Transplant Surgery, Medical College of Wisconsin, Milwaukee, USA

**Keywords:** liver transplant, id critical care, interventional radiology, critical care, spontaneous bleeding, end stage liver disease

## Abstract

Background: Patients with end-stage liver disease (ESLD) are at increased risk for hemorrhage and spontaneous retroperitoneal hematoma (sRPH) and also carry a high mortality rate. We sought to review the natural history of sRPH in patients with ESLD at a single center.

Methods: All patients admitted to a single transplantation intensive care unit (TICU) at Froedtert and the Medical College of Wisconsin Transplant Center between June 2016 and August 2018 were retrospectively reviewed. Six ESLD patients with sRPH were studied. Clinical outcome measures were liver disease severity, sRPH treatment, and patient survival.

Results: Six patients were included, four male and two female patients, with a median age of 56.5 years (range 30-67 years). All had alcohol-induced liver cirrhosis. The median Model for End-Stage Liver Disease (MELD) score at the time of sRPH diagnosis was 40 (range 30-43). The most commonly identified source of bleeding was from lumbar arteries. One patient had recurrent bleeding after embolization and underwent repeat embolization. Five patients died. The median time to death from the diagnosis of sRPH was 7.2 days (range 2-12 days). The patient who survived following embolization had the lowest MELD score.

Conclusion: Critically ill cirrhotic patients with sRPH have a significant mortality rate. Embolization is successful, albeit seldom. This is the largest retrospective series of sRPH in cirrhotic patients in the literature.

## Introduction

Spontaneous retroperitoneal hematoma (sRPH) is a very rare entity and has been reported almost exclusively in patients receiving anticoagulant medication or hemophiliacs. However, sRPH is frequently occult and under-recognized by clinicians, even though it is a significant cause of morbidity and mortality [1,2]. The retroperitoneum contains numerous organs and important tissues; due to its extremely vascular nature, it can be a location of severe bleeding and develop large hematomas caused by trauma, surgical or endovascular interventions in the area [1,2]. Computed tomography (CT) scans with or without contrast enhancement are essential for making a diagnosis of sRPH; the physical exam is usually nondiagnostic, and they are not visible on conventional abdominal plain film [1].

Liver transplantation is a well-established, effective treatment for end-stage liver disease [3]; the number of potential recipients identified for liver transplants is growing, despite the fact that the number of donors is either remaining constant or falling [3]. Because of this, waiting periods are getting longer, contributing to an increase in the morbidity and mortality rates that occur prior to transplantation [3]. Liver cirrhosis is associated with increased risk of bleeding, frequently from portosystemic varices; sRPH has been rarely reported in cirrhotic patients and is associated with high mortality due to the risk of uncontrolled bleeding and acute-on-chronic hepatic failure [4,5].

Due to the limitation of the current report of this lethal disease, there is no available guideline about the management of the clinical practice of sRPH. In this high-risk patient population, there are no clear guidelines for sRPH management, and past approaches have ranged from supportive care alone to interventional radiology or, rarely, surgery [4,6-12].

To the best of our knowledge, this is the biggest number of sRPH ever documented in the scientific literature from a single institution. The goal of this study was to review the diagnosis, management, and clinical outcomes of sRPH in cirrhotic patients at a tertiary-care transplant center specializing in the critical care of patients with end-stage liver disease awaiting liver transplant and we hope to demonstrate our approach to such a rare entity as a guide for other physicians dealing with such rare cases. 

## Materials and methods

The study was conducted at the Transplant Intensive Care Unit (TICU) at the Froedtert and the Medical College of Wisconsin Transplant Center. The study was approved by the Institutional Review Board at the Medical College of Wisconsin.

We retrospectively evaluated the records of the patients with files between June 2016 and August 2018 from our digital medical records system and PACS (Picture Archiving and Communication System). We reviewed all patients at our TICU, diagnosed with sRPH, and received emergency angiographic examination and embolization.

Overall, the study population included six patients. Clinical and laboratory data variables pertaining to the severity of liver disease, such as hemoglobin, activated partial thromboplastin time (aPTT), international normalized ratio (INR), and intensive care acuity at the time of sRPH diagnosis, were collected. Each patient’s clinical course, from diagnosis of sRPH through treatment interventions and outcomes, was reviewed in detail.

All patients had a CT done as a diagnostic technique, with or without contrast, depending on the urgency of their clinical assessment. The location of the hematoma and its extension into the iliopsoas muscle or retroperitoneal region were evaluated by the on-call radiologists. The development of pseudoaneurysms or indications of bleeding was assessed. Contrast medium extravasation was used to localize the site of active bleeding.

## Results

Patient characteristics

The clinical characteristics of the six patients with liver cirrhosis and sRPH are summarized in Table 1. The median age was 56.5 years (range 30-67 years), and four of the six patients were male. All patients had de-compensated liver cirrhosis due to alcohol: five patients had intractable ascites, and three had varices on endoscopy. The median Model for End-Stage Liver Disease (MELD) score at the time of sRPH diagnosis was 40 (range 30-43). Within 30 days before the onset of sRPH, a blood culture-positive infection was diagnosed in two patients, gastrointestinal bleeding in three patients, and an acute portal vein thrombus in one patient. All patients manifested a leukocytosis consistent with their acute inflammatory or infectious condition. Laboratory coagulation tests were all abnormal, including severe thrombocytopenia defined by platelets count <50,000/mL in all patients and below-quantifiable fibrinogen levels defined by <200mg/dL in three patients. All patients were critically ill before the diagnosis of sRPH: three required continuous vasopressor infusions, all mechanically ventilated, and all received continuous renal replacement therapy due to anuric acute kidney injury.

**Table 1:** Clinical characteristics of six patients with decompensated cirrhosis who developed spontaneous retroperitoneal hematomas M, male; F, female; MELD, model for end-stage liver disease; NA, not available (no prior endoscopy); WBC, white blood cell count; GI, gastrointestinal; INR, international normalized ratio.

Patient	1	2	3	4	5	6
Age/sex	62/M	51/M	66/M	67/F	51/F	30/M
Etiology of cirrhosis	Alcohol	Alcohol	Alcohol	Alcohol	Alcohol	Alcohol
MELD	40	40	43	41	30	38
Ascites	Yes	Yes	Yes	Yes	No	Yes
Varices	Yes	NA	NA	No	Yes	Yes
30-day history of infection	No	No	No	No	Yes	Yes
WBC, x10^3^/μL	16	31	23	18	23	21
Bleeding and Coagulation						
30-day history of thrombus	No	No	No	No	No	Yes
30-day history of GI bleeding	Yes	No	Yes	Yes	No	No
INR	2.1	2.2	2.6	2.6	1.4	1.6
Platelet count, x10^3^/μL	35	36	27	24	25	52
Fibrinogen level, mg/dL	<80	109	<80	<80	93	130
ICU Support						
Vasopressor support	Yes	Yes	No	Yes	No	No
Mechanical ventilation	Yes	Yes	Yes	Yes	Yes	Yes
Continuous renal replacement therapy	Yes	Yes	Yes	Yes	Yes	Yes

Diagnosis and treatment of sRPH

**Figure 1:**
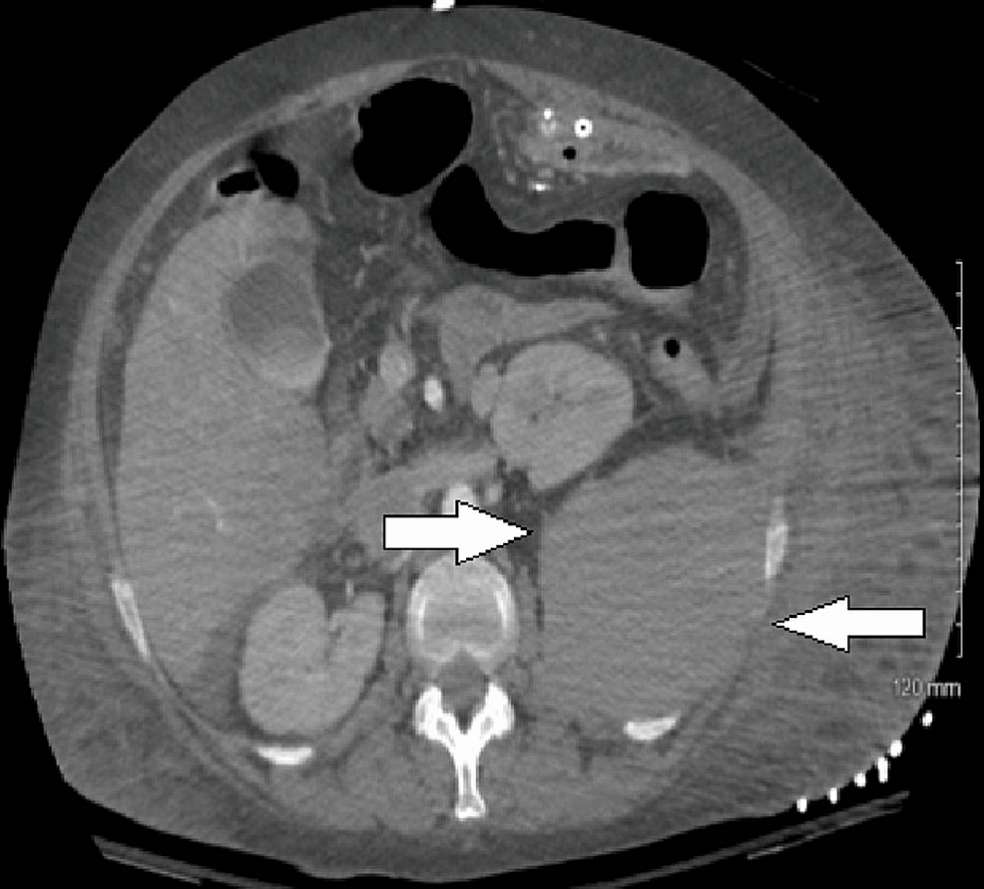
CT image showing spontaneous left retroperitoneal hematoma (arrows)

**Figure 2:**
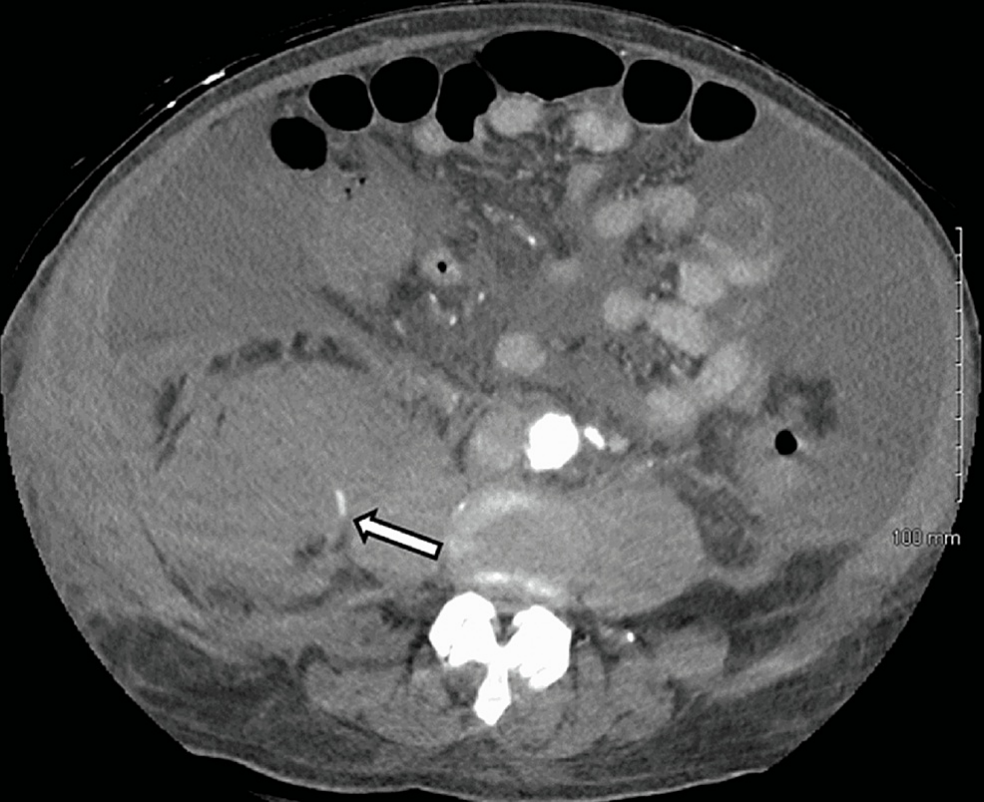
CT angiogram showing contrast extravasation (arrow) within a large right retroperitoneal hematoma, indicating active bleeding

None of the patients were on antiplatelets or anticoagulation therapy, and none of them underwent any invasive procedure or had trauma before the diagnosis of sRPH. In the absence of gastrointestinal or other obvious anothering, an unexplained drop in hemoglobin with only partial or transient response to transfusion, often accompanied by relative hypotension, prompted further workup for occult bleeding. Due to acute renal failure or the urgency of the situation, a non-contrast CT scan was the initial diagnostic study in four patients, and all showed a unilateral sRPH (Figure 1). One of these four underwent a follow-up CT angiogram (CTA). The remaining two patients had a contrast CT as their initial imaging study. Two of these three contrast scans showed extravasation (Figure 2).

**Figure 3:**
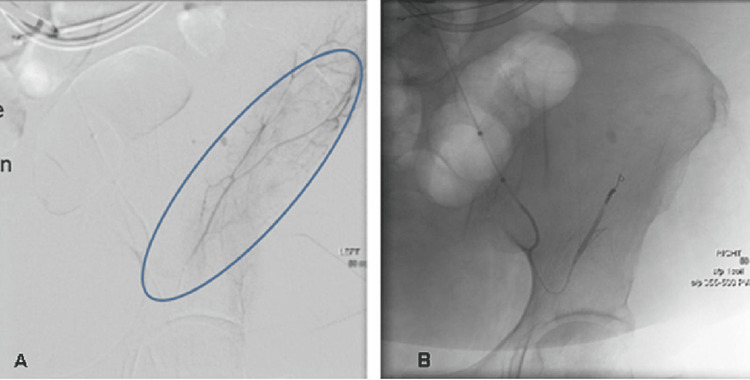
Conventional angiogram showing (A) contrast extravasation (circled) from a branch of the left deep circumflex iliac artery, which was controlled with (B) coil and particle embolization

Once the diagnosis of sRPH was obtained, interventional radiology (IR) was consulted in four cases (Table 2), and all four patients underwent conventional angiography. Three patients demonstrated contrast extravasation, which was embolized (Figures 3A, 3B). All three patients had a bleeding lumbar artery, and one of them also had bleeding from a branch of the deep circumflex iliac artery. Only one patient who did not show contrast extravasation underwent prophylactic embolization of the L3 and L4 lumbar arteries ipsilateral to the sRPH seen on CT. Immediate post-embolization angiography in all patients showed no residual active bleeding.

**Table 2:** Diagnosis, management, and outcome of spontaneous retroperitoneal hematoma in six patients Pt, patient; CT, computed tomography; sRPH, spontaneous retroperitoneal hematoma; L, lumbar; a, artery; CTA, CT angiogram; NA, not applicable.

Pt	Initial diagnostic imaging modality and findings	Source of Bleeding	Intervention performed	Outcome
1	Noncontrast CT: Right sRPH	Extravasation from right L4 a.	Gelfoam embolization	CTA 6 days post-embolization showed enlarging sRPH, punctate foci of extravasation à Recurrent right L4 a. and new right deep circumflex iliac a. bleeding treated by glue embolization. Expired 10 days after the diagnosis
2	Noncontrast CT: Left sRPH	No extravasation	Prophylactic gelfoam embolization of left L3 and L4 aa.	Noncontrast CT 6 days post-embolization showed decrease in size of sRPH Expired 6 days after the diagnosis
3	Noncontrast CT: Right sRPH	NA	NA	Expired 2 days after diagnosis
4	Contrast CT: Bilateral psoas hematomas with contrast extravasation	Extravasation from left L4 a.	Particle embolization	Expired 7 days after the diagnosis.
5	Contrast CT: Left psoas and iliacus hematomas, no extravasation	Extravasation from left L3 and deep circumflex iliac aa.	Coil and particle embolization	CTA 2 days post-embolization showed stable left sRPH, no extravasation. Patient survived
6	Noncontrast CT: Right sRPH	NA	NA	Noncontrast CT 5 and 11 days after diagnosis showed stable sRPH Expired 6 days after diagnosis

Two patients did not receive an IR consultation. One patient’s rapid clinical deterioration and overall moribund condition led to death two days after sRPH diagnosis. While the second (patient no. 6) underwent repeat imaging upon transfer to our facility with the diagnosis of sRPH, which showed a stable-sized hematoma.

Outcomes

Patients continued to require red blood cell transfusion after embolization. Three patients had a follow-up CTA. One (patient no. 1) demonstrated recurrent bleeding from the gel foam-embolized L4 lumbar artery, new bleeding from a branch of the right deep circumflex iliac artery, and interval enlargement in the ipsilateral sRPH. This patient was treated with glue embolization, and post-embolization angiography showed no residual extravasation. Another follow-up CTA showed new punctate foci of contrast extravasation but no interval changes in the size of the sRPH, so no other intervention was performed. The last CTA showed no interval size change of the sRPH and no contrast extravasation. The fourth patient had a non-contrast CT done three days post-embolization for an unrelated reason, which showed an interval decrease in the size of the sRPH.

One patient developed a 1-cm left femoral pseudoaneurysm following the initial embolization procedure. It was incidentally diagnosed six days later on follow-up CTA, but the patient expired the following day without intervention. The remaining five patients had no procedure-related complications.

Of these six critically ill patients with decompensated cirrhosis and sRPH, five died at a median of 7.2 days (range 2-12 days) from diagnosis. The sole survivor with the lowest MELD score of 30 (patient no. 5) was transferred out of the ICU 30 days after embolization and was discharged from the hospital 22 days later and still alive after four years of follow-up. 

## Discussion

Patients with decompensated liver cirrhosis have thrombocytopenia, platelet dysfunction, lower clotting factors, and bleeding diathesis [13,14]. While these factors may contribute to intramuscular and retroperitoneal hematomas, bleeding rarely starts without an extrinsic cause, such as trauma, anticoagulation therapy, hemophilia, and other congenital or acquired coagulopathies unrelated to liver disease, surgical or endovascular procedures, or vascular lesions. Multiple reports have described spontaneous intramuscular or retroperitoneal bleeding related to anticoagulation and other systemic diseases [2,15], but few cases have been reported in patients with liver cirrhosis [4,7-9,16].

All patients in this series had alcoholic cirrhosis, similar to prior studies showing a strong relationship between sRPH and alcoholic liver disease [4,6]. Takamura et al. presented three cases, reviewed 21 case reports in the literature of intramuscular or retroperitoneal bleeding in patients with liver cirrhosis, and found that alcohol consumption was the primary cause of liver cirrhosis in 72% of patients [4]. Mechanisms by which alcoholic liver disease may increase the risk of bleeding beyond the hyperfibrinolysis, thrombocytopenia, and reduction in coagulation factors inherent to cirrhosis [14], include blood vessel wall fragility and promotion of atherosclerosis [10,17,18], inhibition of platelet adhesion in response to fibrinogen [19], reduced platelet activation [20]. A dose-dependent reduction in platelet aggregation induced by extravasation [20,21].

Both pro-coagulant and anti-coagulant changes are seen in liver cirrhosis, prompting some to suggest that the elevated risk of bleeding may be better explained by comorbid conditions that favor hemorrhages, such as portal hypertension, endothelial dysfunction, bacterial infection, and renal failure [14,22-24]. All patients in this series manifested severe portal hypertension and acute renal failure; two patients had an infection within 30 days prior to sRPH. Another indicator of bleeding risk and poor outcome in this rare diagnosis may be the MELD score, which has been shown to predict mortality in alcoholic hepatitis [25], and rebleeding risk and prognosis after acute variceal hemorrhage [26,27]. Notably, this series reports the highest MELD scores in the literature of sRPH patients, and the sole survivor was also the patient who had a MELD score of 30, the lowest of the group.

The diagnosis was made by a CT scan and depended on a high index of clinical suspicion. Although the use of intravenous contrast allows visualization of extravasation and, therefore, active bleeding, even a non-contrast CT showing sRPH in a patient with clinical manifestations of acute bleeding (e.g., tachycardia, hemodynamic instability, and transient or non-response to transfusion, etc.) warrants IR consultation and possibly intervention. The most common source of bleeding was a lumbar artery, consistent with reports of sRPH in both cirrhotic patients and other patient populations [2,4,8,11,15,28]. Therefore, we recommend a targeted approach to interrogate and embolize lumbar arteries ipsilateral of a known sRPH. Three out of four patients who underwent IR angiogram and embolization successfully achieved clinical hemostasis after the initial procedure, defined by stable or smaller sRPH on repeat imaging. Clinical hemostasis was also demonstrated in one of the two patients who did not receive IR consultation: sRPH had been diagnosed in this patient prior to transfer to our hospital, and a repeat CT scan immediately after the transfer, five days after diagnosis, showed interval stability of the sRPH. One patient became too clinically unstable for IR intervention, which underlines the importance of early intervention once the diagnosis is made.

The optimal management of sRPH in cirrhotic patients remains undefined, and mortality remains high. The mortality rate in our series was 83%, which was greater than the 71% mortality rate reported in the literature review of 24 case reports done by Takamura et al. [4]. Importantly, embolization may be associated with improved survival [28]. In the Takamura et al.'s review, although only four patients were treated with embolization, two survived [4]. Other case reports have described successful embolization of sRPH in end-stage liver disease patients [8,11] with survival to discharge [8]. Despite the critical acuity of patients in this series, one of the patients who underwent successful embolization did survive ICU and hospital discharge.

This retrospective review is limited by the small sample size and individualized care that each patient received, factors inherent in the rarity of this diagnosis. Differing approaches to the sRPH diagnosis are reported here outside of the larger context of the patient's comorbidities. The quality of the data depended on the accuracy of the medical record.

## Conclusions

sRPH is associated with significant mortality risk in critically ill patients with end-stage liver disease. Diagnostic CT may be done with or without intravenous contrast, as a conventional angiogram ahead of embolization will further localize the source of bleeding, often from ipsilateral lumbar arteries. Embolization has a poor success rate, although it is crucial to note that it is not completely unsuccessful. To the best of our knowledge, this is the largest single-center series of cirrhotic patients treated with sRPH that has ever been described in the English literature.
